# Dynamics of Colon Monocyte and Macrophage Activation During Colitis

**DOI:** 10.3389/fimmu.2018.02764

**Published:** 2018-11-27

**Authors:** Gareth-Rhys Jones, Calum C. Bain, Thomas M. Fenton, Aoife Kelly, Sheila L. Brown, Alasdair C. Ivens, Mark A. Travis, Peter C. Cook, Andrew S. MacDonald

**Affiliations:** ^1^Lydia Becker Institute of Immunology and Inflammation, Manchester Collaborative Centre for Inflammation Research, Faculty of Biology, Medicine and Health, Manchester Academic Health Science Centre, University of Manchester, Manchester, United Kingdom; ^2^Institute of Immunology and Infection Research, University of Edinburgh, Edinburgh, United Kingdom; ^3^Medical Research Council Centre for Inflammation at the University of Edinburgh, Queens Medical Research Institute, University of Edinburgh, Edinburgh, United Kingdom; ^4^Faculty of Biology, Medicine and Health, Wellcome Trust Centre for Cell-Matrix Research, Manchester Academic Health Science Centre, University of Manchester, Manchester, United Kingdom

**Keywords:** monocyte, macrophage, colitis, chemokine, IBD

## Abstract

**Background:** Macrophages are pivotal in coordinating a range of important processes in the intestines, including controlling intracellular infections and limiting damaging inflammation against the microbiota. However, it is not clear how gut macrophages, relative to recruited blood monocytes and other myeloid cells, contribute to the intestinal inflammatory milieu, nor how macrophages and their monocyte precursors mediate recruitment of other immune cells to the inflamed intestine.

**Methods:** Myeloid cell populations isolated from colonic inflammatory bowel disease (IBD) or murine dextran sulphate sodium (DSS) induced colitis were assessed using flow cytometry and compared to healthy controls. In addition, mRNA expression profiles in human and murine colon samples, and in macrophages and monocytes from healthy and inflamed murine colons, were analysed by quantitative PCR (qPCR) and mRNA microarray.

**Results:** We show that the monocyte:macrophage balance is disrupted in colon inflammation to favour recruitment of CD14^+^HLA-DR^Int^ cells in humans, and Ly6C^Hi^ monocytes in mice. In addition, we identify that murine blood monocytes receive systemic signals enabling increased release of IL-1β prior to egress from the blood into the colon. Further, once within the colon and relative to other myeloid cells, monocytes represent the dominant local source of both IL-1β and TNF. Finally, our data reveal that, independent of inflammation, murine colon macrophages act as a major source of *Ccl7* and *Ccl8* chemokines that trigger further recruitment of their pro-inflammatory monocyte precursors.

**Conclusions:** Our work suggests that strategies targeting macrophage-mediated monocyte recruitment may represent a promising approach for limiting the chronic inflammation that characterises IBD.

## Introduction

The gastrointestinal (GI) tract is exposed to the greatest antigenic burden in the body, which poses a significant challenge to the many resident immune cells (1). Macrophages form the largest component of the intestinal mononuclear phagocyte system and play a pivotal role in mediating immune homeostasis (2). The IBDs, including ulcerative colitis (UC) and Crohn's disease (CD), are characterised by a disturbed immunological homeostasis in genetically susceptible individuals, although the exact aetiology is unknown (3–5).

It has recently been shown that murine intestinal macrophages are continually repopulated from circulating blood monocytes, identified by expression of the cell surface markers Ly6C, CCR2, and CD62L (6). However, the functions of mature intestinal macrophages and recruited monocytes are strikingly different. Intestinal macrophages display a “tolerant” phenotype, poorly responsive to toll like receptor (TLR) ligands (vital to avoid triggering inflammatory responses while scavenging commensal bacteria) whilst maintaining a homeostatic environment via IL-10 and PGE_2_ production (7). In contrast, murine colon monocytes are pro-inflammatory but infrequent in the steady state, increasing during inflammation (2, 8–10).

Previous studies have shown the accumulation of human CD14^Hi^ or CD14^+^HLA-DR^Int^ cells in the GI tract lamina propria (LP) during inflammation (2, 6, 11–14). Results from murine studies (2) combined with the observation of radiolabelled blood monocytes in the inflamed intestinal mucosa of IBD patients (15), indicate that increased LP inflammatory mononuclear cells are a result of monocyte recruitment, rather than expansion of a tissue resident macrophage population. Therefore, as monocytes and macrophages are considered potential cellular targets for therapy in limiting damaging host responses in intestinal disease (16), there is an urgent need to improve the resolution of these events in the mucosal tissue.

The surface phenotype of immune cells is dependent on their location in the GI tract, which is critical when interpreting previous studies. For example, both small intestine and colon macrophages display high expression of MHC-II, CD163, and (in mice) CX3CR1, while colon macrophages express greater levels of acid phosphatase, CD40, CCR5, and formyl peptide receptor (17–19). Similarly a recent seminal study of genotype-phenotype in IBD highlighted how disease location in the GI tract strongly correlates with genetic susceptibility (20). As such, the mechanisms underlying intestinal inflammation are likely to vary depending on the GI tract location, for example small intestine vs. large intestine. Human data thus far has been largely limited to combined surgical resections from both large and small intestine from IBD patients or healthy patients undergoing resection for another indication (such as urostomy formation) (2, 12, 14, 21, 22). Therefore, it is not clear which facets of the colon immune response contribute to the inflammatory phenotype previously reported for IBD.

We have utilised targeted human colon biopsies to assess monocyte:macrophage balance during colon inflammation in patients with IBD vs. healthy controls, alongside complementary work using the murine DSS model of colitis. A combination of multi-parameter flow cytometry, qPCR, and mRNA microarrays were used to define disruption of the resting monocyte:macrophage balance, with influx of CD14^+^HLA-DR^Int^ cells or Ly6C^Hi^ monocytes in humans and mice, respectively.

Whilst monocytes are thought to be important inflammatory cytokine producing cells, their contribution to the inflammatory milieu relative to other myeloid cells in the gut is not known. We show that in murine colitis, colon monocytes represent the key myeloid source of IL-1β. Although TNF^+^ monocytes also expanded in response to DSS, overall TNF^+^ myeloid cells were less frequent than IL-1β^+^ cells. In addition, blood monocytes produced significantly greater IL-1β but not TNF during DSS colitis when stimulated with LPS, suggesting this pro-inflammatory potential is enhanced even before leaving the blood.

Further, we have defined for the first time transcriptomic differences associated with monocyte-macrophage maturation and inflammation in murine colon macrophage and monocyte populations from steady state and DSS colitis. Intriguingly, the genes most up-regulated comparing monocytes to macrophages were conserved in both steady state and inflammation and represented chemokines that promote monocyte recruitment, including *Ccl7, Ccl8*, and *Ccl12* (9, 23). Lastly, we show that *CCL7* and *CCL8* were also markedly elevated in human biopsy material from active IBD.

Together, this suggests that in both humans and mice the tolerogenic status quo of steady state macrophages is overturned during colon inflammation to promote recruitment of their potent pro-inflammatory monocyte precursors.

## Materials and methods

### Mice

Female C57BL/6 wild-type (WT) mice aged 12–22 weeks were maintained under specific pathogen free conditions (SPF) at the University of Manchester, in compliance with the United Kingdom Animals (Scientific Procedures) Act 1986. CX3CR1^+gfp^ mice were maintained under SPF conditions at the Central Research Facility, University of Glasgow (24).

### DSS model

Mice received 2% DSS salt (reagent grade MW 36,000–50,000 kDa; MP Biomedicals, Solon OH) *ad libitum* in sterile drinking water for 6 days, as described previously (2).

### Patients and tissues

#### Flow cytometry

Thirteen IBD (UC or CD) patients undergoing colonoscopy for disease assessment had biopsies taken from endoscopically inflamed (active IBD; 6 samples from 6 patients) or non-inflamed (quiescent IBD; 10 samples from 7 patients) colon in addition to 4 patients attending for colonoscopy for assessment of IBS symptoms (healthy controls; 4 samples from 4 patients) for flow cytometry analysis. In those patients with quiescent IBD that had more than one biopsy analysed, these were from discrete gut segments >10 cm apart as described in Table 1. Healthy control patients had a normal colonoscopy, had no other past medical history and were not ultimately diagnosed with any GI tract pathology (Table 1).

**Table 1:** Flow cytometry colon biopsy sample patient demographics.

**Disease category**	**IBD**	**Patient ID #**	**Sex**	**Age**	**Montreal classification**				**Biopsy location**	**Drugs**	

					**Crohn's disease**			**UC**		**IM**	**Other**
					**Age**	**Location**	**Behaviour**			
Normal	–	1	M	42					Sigmoid	
Normal	–	2	F	39					Sigmoid	
Normal	–	3	F	56					Sigmoid	
Normal	–	4	F	47					Sigmoid	
Quiescent	CD	5	M	41	A1	L3	B1		Transverse	AZA, IFX
Quiescent	CD	6	F	35	A2	L3	B1		Transverse	
Quiescent	CD	6	F	35	A2	L3	B1		Rectum	
Quiescent	CD	7	M	42	A1	L3	B2		Transverse	
Quiescent	CD	7	M	42	A1	L3	B2		Sigmoid	
Quiescent	CD	8	F	27	A2	L3	B1		Transverse	
Quiescent	CD	8	F	27	A2	L3	B1		Sigmoid	
Quiescent	UC	9	F	65				E1	Rectum	
Quiescent	UC	10	M	45				E1	Rectum	
Quiescent	UC	11	F	27				E2	Sigmoid	
Active	CD	12	M	49	A1	L3	B2		Ascending	
Active	CD	13	M	27	A2	L2	B1		Ascending	MTX, ADA
Active	CD	14	M	52	A2	L3	B2		Transverse	AZA
Active	CD	15	M	30	A1	L3	B1		Transverse	
Active	UC	16	M	27				E3	Rectum	AZA	5′ASA
Active	UC	17	F	27				E2	Rectum	

*13 IBD (UC or CD) patients undergoing colonoscopy for disease assessment had biopsies taken from endoscopically inflamed (active IBD; 6 samples from 6 patients) or non-inflamed (quiescent IBD; 10 samples from 7 patients) colon in addition to four patients attending for colonoscopy for assessment of IBS symptoms (healthy controls; 4 samples from 4 patients) for flow cytometric analysis. Patient records were reviewed for demographic, IBD location and disease behaviour by Montreal classification. IM, Immunomodulatory drugs; MTX, methotrexate; ADA, adalimumab; IFX, infliximab; 5′ASA, 5′aminosalicylic acid. Montreal classification for CD: A1, age at onset < 16 years; A2, between 17 and 40 years; A3, >40 years. L1, ileal disease location; L2, colonic disease location; L3, ileocolonic disease location. B1, inflammatory phenotype; B2, stricturing phenotype; B3, penetrating phenotype. Montreal classification for UC: E1, proctitis; E2, rectosigmoid disease; E3, disease proximal to the splenic flexure. CD, Crohn's disease, UC, ulcerative colitis. # Patient ID is the identifier allocated to each individual providing tissue, such that donors that gave more than 1 sample can be identified*.

#### RT-qPCR

A separate group of 28 IBD (UC or CD) patients undergoing colonoscopy for disease assessment had biopsies taken from endoscopically inflamed (active IBD; 19 samples from 17 patients) or non-inflamed (quiescent IBD; 12 samples from 11 patients) colon in addition to eight patients attending for colonoscopy for assessment of IBS symptoms (healthy controls; 12 samples from 8 patients) for RNA extraction and RT-qPCR analysis. Where more than one biopsy was analysed, these were from discrete gut segments >10 cm apart as described in Supplementary Table 1. Healthy control patients had a normal colonoscopy and had no other past medical history, with only one patient ultimately given a diagnosis of IBS (Supplementary Table 1). Montreal classification and drug information were derived from review of patient records (Table 1; Supplementary Table 1).

### Isolation of LP cells

LP cells were obtained from mouse colon by enzymatic digestion as described previously (2). Human colon biopsies were incubated in Hank's balanced salt solution (HBSS), 1% Penicillin-streptomycin (PenStrep) (Sigma-Aldrich), G418 (Melford) and 1 mM DTT (Sigma-Aldrich) for 15 min followed by three 15 min washes at 37°C with rotation (200 rpm) with HBSS, PenStrep, G418 and 1 mM EDTA (Sigma-Aldrich) to remove epithelial cells. The tissue was then digested in 1 mg/ml Collagenase A (Roche) in 10% FCS RPMI, 1% PenStrep and G418 with 60 U/ml DNAse I (Sigma-Aldrich) for 1 h in a shaking incubator at 37°C. After this time, the tissue was strained through a 40 μM filter, and cells analysed by flow cytometry or lysed in RLT buffer (Qiagen) for RNA extraction.

### Flow cytometric analysis and sorting of cells

0.5–2 × 10^6^ cells were stained at 4°C as described previously using the antibodies listed in Supplementary Table 2 (6). To detect intracellular cytokines, cells were incubated in complete RPMI (Sigma-Aldrich plus 10% FCS 1% PenStrep) at 37°C in 5% CO_2_ for 3 h in the presence of 1 μl/ml GolgiStop (BD Biosciences). After surface staining, cells were fixed in 1% paraformaldehyde, washed in phosphate-buffered saline (PBS) and permeabilised using Cytofix/Cytoperm (BD Biosciences). After a further stain with PE anti-TNF and FITC anti-IL-1β or isotype controls for 1 h, cells were washed prior to sample acquisition. All stained samples were acquired using a LSRFortessa (BD Biosciences) and analysed using FlowJo software (TreeStar, Ashland, OR). The number of myeloid cells per mouse colon was determined by calculating the proportion of the identified populations (Figure 2A) as a proportion of Live/singlet/intact/CD45^+^/Lineage^−^ cells. The sum of the populations expressed in Figure 2A accounted for >97% of these cells. Myeloid population data was then expressed per mouse colon using the total cell count derived after purification.

Macrophage and monocyte populations were purified using fluorescence-activated cell sorting (FACS) for subsequent gene expression analysis. In the steady state, CD45^+^CD11b^+^CX3CR1^Int^Ly6C^+^MHC-II^−^ monocytes and CD45^+^CD11b^+^CX3CR1^Hi^Ly6C^−^MHC-II^+^F4/80^+^ macrophages were purified from CX3CR1-GFP mice as previously described (2). In DSS colitis, CD45^+^Ly6G^−^Siglec-F^−^CD11b^+^Ly6C^+^MHC-II^+/−^ monocytes and CD45^+^Ly6G^−^Siglec-F^−^CD11b^+^Ly6C^−^MHC-II^+^F4/80^+^ macrophages were sorted from C57BL/6 mice that had been exposed to DSS for 6 days.

### Murine blood monocyte isolation

Blood was obtained by cardiac puncture and combined with 200 μl of 2 mM EDTA 3% FCS in PBS to prevent coagulation. Serum was aspirated after pelleting of whole blood at 500 G, 4°C for 5 min before re-suspension of haematocrit in 5 ml red cell lysis buffer (Sigma-Aldrich) for 7 min to lyse red blood cells. 2 × 10^6^ cells were incubated for 3 h at 37°C, 5% CO_2_ with 1 ug/ml LPS (Sigma-Aldrich) before surface/intracellular staining for CD68, CD11b, Ly6C, Lineage markers (NK1.1, CD19, CD3, Ter119, B220), IL-1β and TNF.

### mRNA microarray and quantitative PCR (qPCR)

For murine colon samples or sorted cell populations total RNA was purified using RNeasy mini kits (Qiagen), as previously described (25). For microarrays, RNA was labelled using TotalPrep RNA amplification kits (Life Technologies) and hybridised with Illumina MouseWG-6BeadChip arrays with five biological replicates from one experiment of FACS-purified macrophages and monocytes. The microarray data from the naïve mice were retrieved from the GEO data set GSE84764 (26). All analyses were conducted in R using Bioconductor. Pairwise group comparisons were undertaken using linear modeling. Subsequently, empirical Bayesian analysis was applied, including vertical (within a given comparison) *P*-value adjustment for multiple testing, which controls for false-discovery rate, using the limma Bioconductor package.

For RT-qPCR of colon tissue, RNA was extracted using RNeasy mini kits (Qiagen) and complementary DNA was generated using GoScript reverse transcriptase kits (Promega). Relative quantification of genes of interest was performed by qPCR analysis using QuantStudio 12 Flex Real Time OCR system, with Fast SYBR® Green Master Mix (Life Technologies), compared with a serially diluted standard of pooled complementary DNA. Transcript expression was normalised to the housekeeping gene *GAPDH*, which did not vary significantly during inflammation (data not shown). Primers are listed in Supplementary Table 3.

### Statistical analysis

Statistical analyses were carried out using GraphPad Prism v.7 or JMP v.12 (SAS Institute). The data were checked to confirm normality and that groups had equal variance. One-way analysis of variance (ANOVA) with Tukey's multiple comparison tests was employed to determine significant differences between sample groups. Results from these tests were reported as significant if *P* < 0.05, with results from these tests shown as mean ± SEM. For some experiments statistical analysis was carried out using JMP, in which case data were analysed using three-way full-factorial fit models to assess effects, such as “genotype,” “treatment,” and “experiment” on the response variable of interest. This allowed the interaction between effects to be taken into account in addition to their impact on the response variable, which enabled experimental repeats to be pooled increasing the power of the analysis (27). The least squares mean results table from the three-way full-factorial analysis was used to test the contrast between specific experimental groups using a joint F-test. A difference between experimental groups was taken to be significant if the *P*-value (Prob > F) was < 0.05, with results in graphs shown as least squares mean ± SEM.

### Ethical considerations

All samples were obtained according to the principles expressed in the Declaration of Helsinki and under local ethical guidelines and approved by the North West National Research Ethics Service (reference number 15/NW/0007). All patients provided written informed consent for the collection of tissue samples and subsequent analysis.

## Results

### Active human colon IBD is characterised by recruitment of CD14^Hi^HLA-DR^Int^ cells

**Figure 1:**
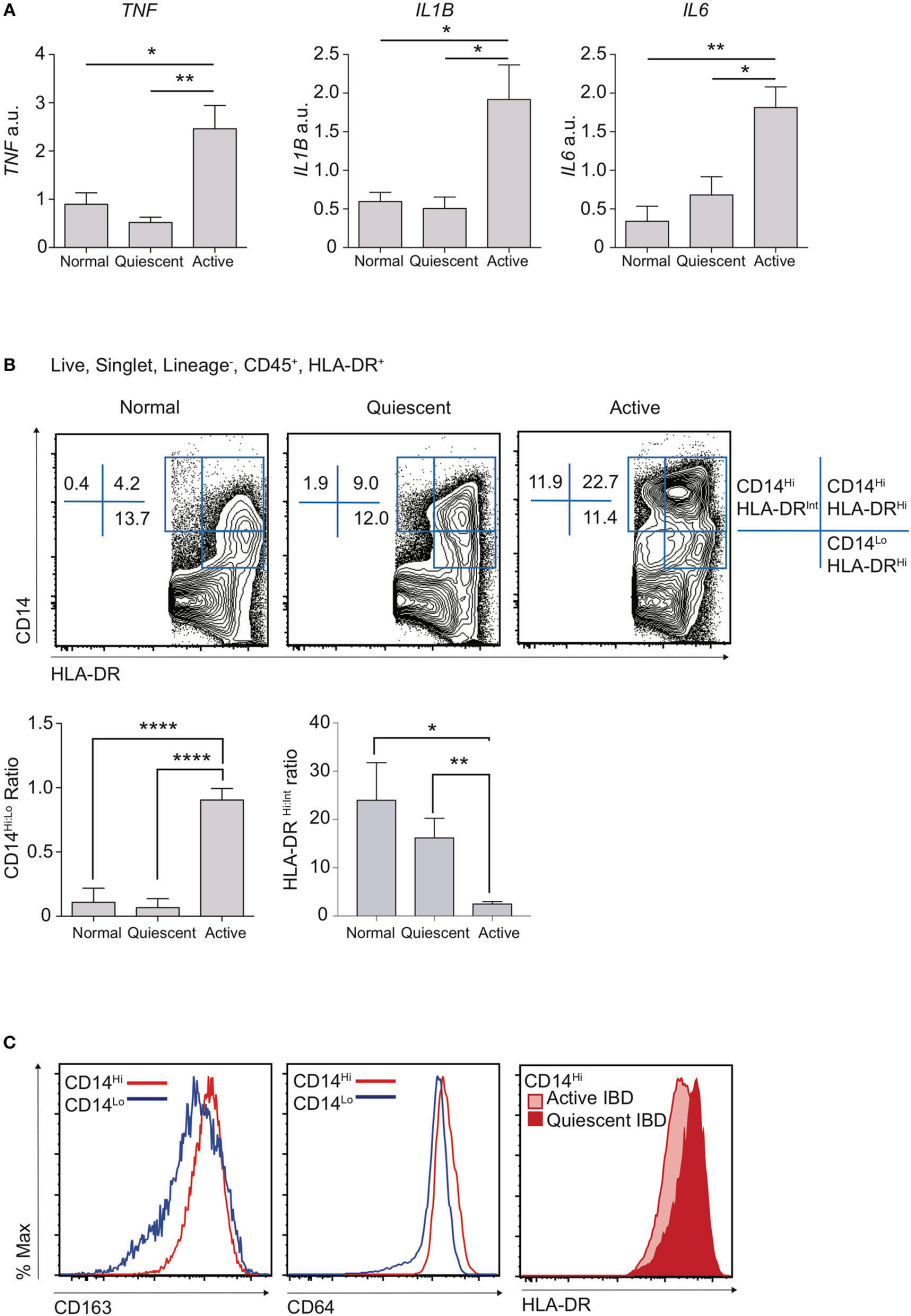
Disruption of resting CD14^Hi^:CD14^Lo^ populations in active IBD. Patients with IBD had colonic biopsies taken from macroscopically inflamed (active IBD) or non-inflamed (quiescent IBD) areas and compared to sigmoid colon biopsies from healthy controls. **(A)** mRNA isolated from biopsy samples was analysed by qPCR for expression of IL1B, IL6, and TNF with mean values relative to GAPDH shown (12 healthy control, 12 quiescent IBD, and 19 active IBD biopsy samples from 8, 11, and 17 healthy control/patients, respectively were analysed in three separate experiments, representative data shown) (Supplementary Table 1). **(B,C)** Colon lamina propria cells were isolated from an additional 4 healthy control, 10 quiescent IBD and 6 active IBD biopsy samples (from 4, 7, and 6 healthy control/patients, respectively) and analysed by flow cytometry (Table 1). **(B)** Live, singlet, Lineage^−^ (CD3, CD19, CD20, CD56) cells were then assessed for the expression of CD14, CD45, CD11c, CD64, CD163, and HLA-DR. Ratios of CD14^Hi^ to CD14^Lo^, Lineage^−^CD45^+^ and CD14^Hi^ HLA-DR^Hi^ to HLA-DR^int^ cells were compared in biopsy samples. **(C)** Histograms of CD163 and CD64 in CD14^Hi^ and CD14^Lo^ healthy controls (left and middle panel) and HLA-DR in CD14^Hi^ populations from active and quiescent IBD (right panel) *P < 0.05, **P < 0.01, ****P < 0.001. a.u., arbitrary units.

A dominant feature of IBD is damaging local production of pro-inflammatory cytokines (28). Indeed, long-term administration of monoclonal antibodies against TNF still forms the mainstay of treatment for patients with complicated disease (29, 30). Polymorphisms in *IL1B, IL6*, and *TNF* have all been associated with predisposition to IBD, suggesting variation in the ability to regulate pro-inflammatory cytokine production is associated with disease susceptibility (31). We therefore used qPCR to assess the mRNA expression “signature” of key inflammatory genes in targeted colon biopsy samples of macroscopically inflamed or quiescent areas from patients with IBD, or from healthy controls (Figure 1A; Table 1). Consistent with expectation, mRNA transcripts for *TNF, IL1B*, and *IL6* were significantly increased in active IBD vs. quiescent IBD or healthy control patient samples, confirming that these cytokines are a hallmark of disease activity (Figure 1A) and this was unaffected by drug treatment.

We hypothesised that the balance of tolerogenic steady state macrophages vs. pro-inflammatory monocytes likely defines resting and inflamed human colon tissue, particularly given that these populations have been shown to be in continuum in murine studies (2, 6). We therefore compared proportions of macrophages and monocytes in inflamed vs. quiescent colon biopsies. Though distinction between intestinal macrophages and monocytes is more challenging in humans than in mice, recent work has shown that there is significant conservation of markers that also define murine intestinal macrophages, such as CD14, CD64, CD68, MHC-II, and CD163 (2, 13, 18, 31). Indeed, as in mice where maturity of resident colon macrophages is inversely associated with expression of Ly6C, human macrophages are thought to express low levels of CD14 (CD14^Lo^) whilst there also exists a smaller CD14^Hi^ subset that is more heterogeneous for expression of CD163, HLA-DR, and CD209 and may represent the Ly6C^+^ monocytes in mice (2). We therefore used a combination of CD14 and HLA-DR expression to define human monocyte/macrophage subsets (Supplementary Figure 1A) and found that, when comparing CD14^+^ populations, CD14^Lo^HLA-DR^Hi^ cells were most frequent in the steady state, with a much lower proportion of CD14^Hi^ cells (Figure 1B). Notably, in line with their designation as part of the macrophage lineage, both these populations expressed high levels of CD64 and CD163 (Figure 1C). We found that patients with active IBD displayed a striking accumulation of CD14^Hi^ cells, compared to both quiescent IBD and healthy control individuals (Figure 1B), and this was unaffected by drug treatment. Notably, whereas CD14^Hi^ cells in healthy control and quiescent IBD samples expressed high levels of HLA-DR, CD14^Hi^ cells found in active IBD had a HLA-DR^Int/Hi^ profile, consistent with a more immature state (Figures 1B,C). In contrast, whilst the proportion of CD14^Lo^ macrophages was largely unchanged in inflammation, the ratio of CD14^Hi^:CD14^Lo^ cells was significantly altered, due to the accumulation of a CD14^Hi^HLA-DR^Int/Hi^ population (Figures 1B,C).

Thus, we have shown in a targeted biopsy dataset from a colon IBD cohort that, analogous to the changes that have previously been described in mice (9), the balance of human colon intestinal CD14^Lo^ macrophages is perturbed in active IBD, favouring the accumulation of CD14^Hi^HLA-DR^Int^ monocyte-like cells.

### Murine colitis is characterised by recruitment of CD11b^+^Ly6C^+^CD11c^−^MHC-II^+/−^ monocytes

**Figure 2:**
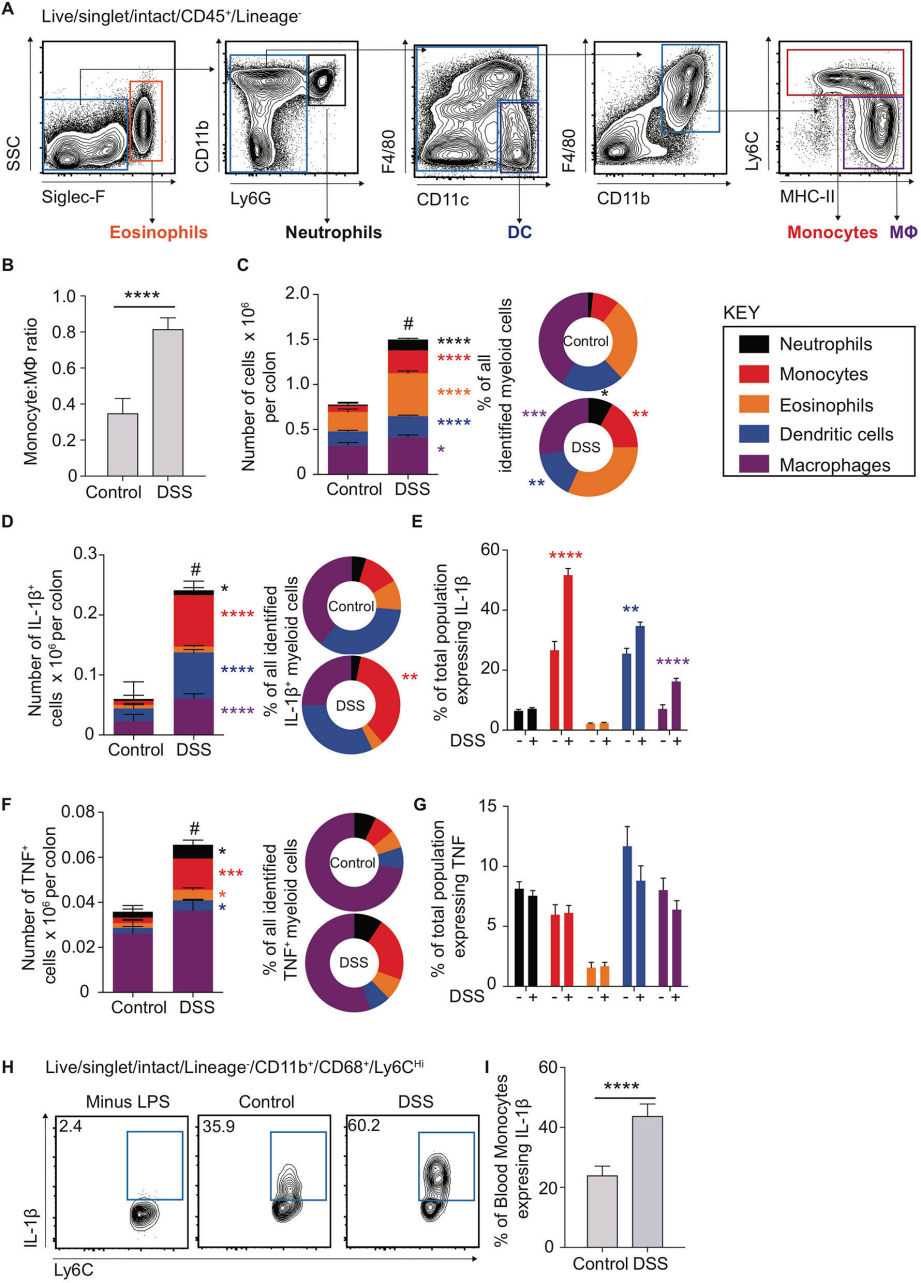
Cytokine production in murine blood and colon monocytes. WT mice received 2% DSS in drinking water (or control drinking water) for 6 consecutive days, colon lamina propria cells and the cellular component of blood was isolated and assessed for the expression of Siglec-F, Ly6G, CD11b, CD11c, F4/80, CD45, and Lineage markers (CD3, CD19, NK1.1, Ter119) (colon) or Ly6C, Lin (NK1.1, CD19, CD3, Ter119, B220), CD11b and CD68 (blood) by flow cytometry. **(A)**. Representative flow cytometry contour plots in day 6 DSS treated WT mice of Live/singlet/Lineage^−^CD45^+^ cells. **(B)**. Least square mean ratio of total number of colon LP Monocytes:Macrophages identified from **(A)** in DSS and drinking water controls **(C)**. The least square mean total number of cells per colon for the populations in **(A)** (left) and their relative proportions (right), *n* = 15–25 per group, analysed by linear regression of six independent experiments. The least square mean total number of cells per colon **(D)**-left and **(F)**-left, proportion of all, **(D)**-right and **(F)**-right, and percentage of total population **(E)** and **(G)** expressing IL-1β **(D)** and **(E)** or TNF **(F)** and **(G)** after 3 h incubation with 1 μl/ml GolgiStop for the populations in **(A)** as assessed by intracellular staining and flow cytometry compared to isotype antibody control, *n* = 12–15 mice per group analysed by linear regression of three independent experiments. **(H)**. Representative flow cytometry plots of blood monocytes isolated from day 6 DSS treated or drinking water controls stimulated with 1 μg/ml LPS and GolgiStop 1 μl/ml for 3 h and assessed for the expression of IL-1β by intracellular staining, with the least mean square % of total blood monocytes **(I)**, *n* = 12–15 mice per group analysed by linear regression of three independent experiments. *P < 0.05, **P < 0.01, ***P < 0.001, ****P < 0.0001, ^#^*P* < 0.0001 for total cell number vs. controls.

To interrogate the molecular mechanisms underpinning the dysregulated CD14^Hi^:CD14^Lo^ axis and cellular sources of IL-1β and TNF in colon inflammation we had observed in human biopsy material, and its contribution to colon inflammation, we employed the use of an established model of murine experimental colitis. DSS colitis is characterised by disruption of the colon epithelial barrier and subsequent inflammation directed towards the infiltrating microbiota (32, 33). This process is dependent on intestinal microbial colonisation and independent of T and B cells, permitting definition of innate immune cell involvement (33, 34) However, the monocyte:macrophage balance and comparative cytokine production during the propagation of DSS colitis is not well-defined, even though these innate cells have been heavily implicated in development of inflammatory pathology in this model (2, 9, 35, 36). Use of multi-parameter flow cytometry (Figure 2A) showed that, similar to healthy human biopsies (Figure 1B), the mouse colon contained a large population of macrophages (defined as F4/80^+^MHC-II^+^CD64^+^CD11b^+^) and a small population of monocytes (Ly6C^+^CD11b^+^MHC-II^+/−^; Figure 2B) (6). This balance was disrupted in DSS-induced colitis, which led to a dramatic increase in monocytes (Figure 2C). Assessment of the entire myeloid cell compartment revealed that neutrophils (CD11b^+^Ly6G^+^) and monocytes underwent the greatest numerical increase (9.2 and 3.7-fold change, respectively), followed by eosinophils (SSC^Hi^Siglec-F^+^, 2.2-fold change) and dendritic cells (DCs) (CD11c^+^F4/80^Lo^CD64^−^, 2.3-fold change). In contrast, the change in macrophage numbers was less dramatic (1.3-fold change) following DSS treatment (Figure 2C—left). Thus, as a proportion of all myeloid cells, inflammation induced an increase in monocytes, neutrophils and eosinophils, alongside reduced proportions of DCs and macrophages, compared to the steady state (Figure 2C—right). This shows that, as we observed in active human IBD, murine colitis is characterised by a striking disruption of the resting monocyte:macrophage ratio (Figures 1B, 2B).

### Colon monocytes in murine colitis express high levels of IL-1β and TNF

The pro-inflammatory cytokines IL-1β and TNF are highly expressed in the mucosa of patients with active IBD (Figure 1A) (37). Further, although mice treated with IL-1β neutralising antibodies display reduced DSS inflammation and expression of IL-6 mRNA transcripts, TNF levels are unaltered (37), indicating that IL-1β may play a more dominant role in promoting DSS colitis than TNF. Recent evidence suggests that perhaps non-monocyte sources of IL-1β may be important in *C. rodentium* infection, as chimeric mice (CCR2^WT^/*Il1b*^−/−^ vs. CCR2^WT^) where 50% of monocytes lack the ability to produce IL-1β (1:1 mix CCR2^WT^/*Il1b*^−/−^) still produce what appears to be equivalent IL-1β in colon LP supernatant to CCR2^WT^ (38). As such, monocyte egress from the blood to the intestines could be hypothesised to stimulate IL-1β production from non-monocyte sources (38). We therefore directly compared *ex vivo* pro-inflammatory cytokine production by monocytes, macrophages and other myeloid cells in steady state and following DSS administration, using flow cytometry to assess intracellular IL-1β and TNF protein levels. There were significant increases in the absolute number of IL-1β^+^ macrophages, DCs, monocytes and, to a lesser extent, neutrophils (Figure 2D). However, monocytes accounted for the largest increase following DSS administration, in both absolute numbers (12.2-fold change) and proportion, to represent 35.7% of all IL-1β^+^ myeloid cells (Figure 2D). Not only were IL-1β^+^ monocytes more numerous in DSS colitis, but the proportion of monocytes expressing IL-1β increased significantly (1.9-fold to 51.7% of all monocytes), which was greater than any other IL-1β expressing myeloid population (Figure 2E). Thus, although there were diverse myeloid sources of IL-1β during DSS colitis, monocytes were the most responsive and prominent IL-1β^+^ population, undergoing the greatest numerical and per cell changes in this setting. As such, the impact of colon monocytes on inflammation during DSS colitis is likely a compound effect of increased per cell IL-1β production along with increased recruitment.

Compared to IL-1β^+^, there were far fewer intestinal myeloid cells that were TNF^+^ in steady state or during DSS colitis (compare Figures 2D,F). Macrophages were the most dominant TNF^+^ population, independent of inflammation, but demonstrated only small changes in total number following DSS administration. As seen with IL-1β secretion, of the TNF^+^ myeloid cell subsets, monocytes expanded the most after DSS administration (5.6-fold change), and increased in proportion to represent 21.1% of all TNF^+^ myeloid cells. However, in contrast to IL-1β, there was no significant increase in the proportion of monocytes that expressed TNF.

### Blood monocytes in murine colitis are conditioned to be pathogenic before arrival into inflamed tissue sites

Given that the proportion of murine colon IL-1β^+^ monocytes increased more dramatically than any other myeloid population in DSS (Figure 2E), we questioned whether this reflected an increased ability of monocytes to respond to an inflamed micro-environment, and/or a result of being primed to be pro-inflammatory systemically prior to arrival in the mucosa. To address this, we cultured monocytes isolated from the blood of DSS treated or control mice *ex vivo* in the presence, or absence, of LPS and assessed their production of pro-inflammatory IL-1β and TNF using intracellular flow cytometry. Ly6C^Hi^CD11b^+^CD68^+^ blood monocytes from DSS treated animals displayed a significantly greater proportion of cells expressing IL-1β, but not TNF (Figures 2H,I; Supplementary Figure 1B), similar to the responses observed from colon monocytes (Figures 2E,G). In addition, neither IL-1β nor TNF were detectable in blood monocytes in the absence of LPS stimulation, suggesting negligible levels of basal cytokine expression.

### Murine colon inflammation changes expression of similar ER stress response genes in both macrophages and monocytes

**Figure 3:**
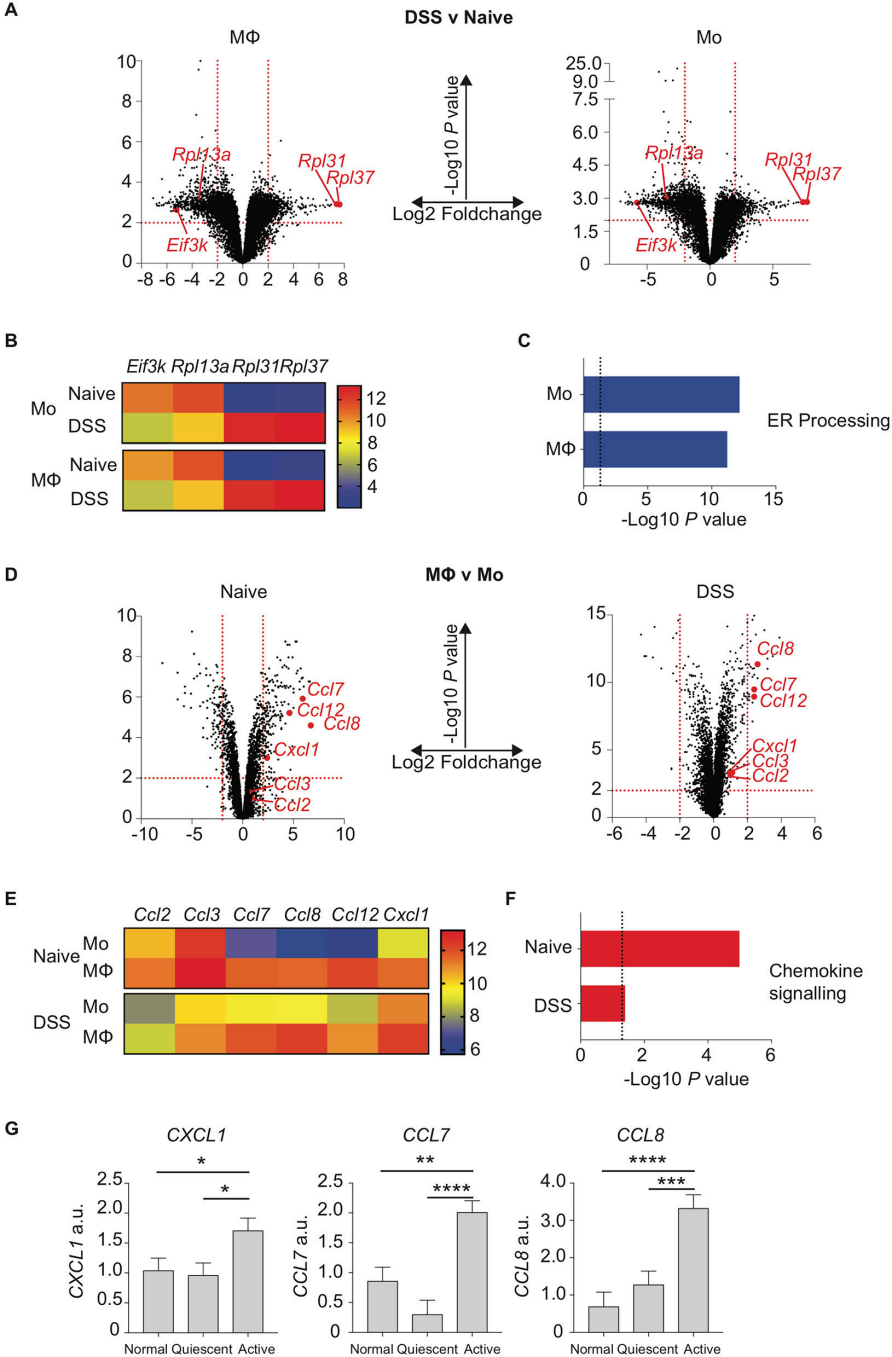
Chemokine expression is increased in inflamed murine colon macrophages and active human IBD. Colon lamina propria macrophage and monocyte populations were isolated and purified by flow cytometry from day 6 2% DSS (CD11b^+^MHC-II^+^Lineage^−^Ly6C^−^ macrophages, CD11b^+^MHC-II^+/−^Lineage^−^Ly6C^+^ monocytes) or naïve mice. RNA was extracted, and gene expression assessed by hybridisation to IlluminaMouseRef6 microarray (2% DSS) or Affymetrix5 chip (Naïve). **(A)**. Volcano plots of Log10 significance and Log2-fold change for gene expression comparisons of DSS vs. naïve macrophages and monocytes. **(B)**. Heat map of selected normalised gene expression from **(A)** (adjusted *P* < 0.01) each individual heatmap represents a biological replicate composed of 2–5 pooled mice. **(C)** KEGG pathway analysis of significant (adjusted *P* < 0.01) pathways from **(A)**. **(D)**. Volcano plots of Log10 significance and Log2-fold change for gene expression comparisons of monocytes vs. macrophages in DSS and steady state. **(E)**. Heat map of selected normalised gene expression from D (adjusted *P* < 0.01) each individual heatmap represents a biological replicate composed of 2–5 pooled mice. **(F)** KEGG pathway analysis of significant (adjusted *P* < 0.01) pathways from **(D)**. **(G)**. Patients with IBD had colon biopsies taken from macroscopically inflamed or non-inflamed areas and compared to biopsies from healthy controls. mRNA isolated from biopsy samples was analysed by qPCR for expression of CXCL1, CCL7 and CCL8 with mean values relative to GAPDH shown (12 healthy control, 12 quiescent IBD, and 19 active IBD biopsy samples from 8, 11, and 17 healthy control/patients, respectively were analysed in three separate experiments, representative data shown) (Supplementary Table 1). *P < 0.05, **P < 0.01,***P < 0.001, ****P < 0.0001. a.u., arbitrary units.

We next sought to understand how the disrupted monocyte:macrophage balance we had identified in both murine and human colitis related to altered gene expression by these key myeloid cells. Colon LP macrophages and monocytes were purified by FACS from control and DSS treated mice, RNA extracted and analysed by transcriptome microarray. Firstly, we looked at the effect of colon inflammation on monocyte and macrophage mRNA expression. By comparing populations from DSS treated vs. naïve mice, we identified 1,446 genes in macrophages (924 down- and 522 up-regulated) and 1,284 genes in monocytes (858 down- and 426 up-regulated) that were significantly altered (Log2-fold change ≥ 2, *P* < 0.01; Figure 3A). Of these, 151 of the 200 most differentially expressed genes (i.e., the largest absolute fold 100 up- and 100 down-changing genes) were the same in both macrophages and monocytes (DSS vs. naïve). This suggested that inflammation confers similar mRNA expression changes in both intestinal macrophages and monocytes. The greatest fold change in gene expression were in mRNA transcripts of ribosomal proteins and ER stress (*Eif3k, Rpl13a, Rpl31, Rpl37*) changes in all of which were similar in both macrophages and monocytes (Figure 3B) (39, 40, 41). Indeed, KEGG analysis showed that the most significant pathway was “protein processing in the ER” which was the down-regulated after DSS in both monocytes and macrophages (Figure 3C).

### Murine colon monocyte to macrophage maturation changes chemokine gene expression irrespective of inflammation

We next looked at the effect of monocyte to macrophage maturation on mRNA expression profiles from naïve or DSS treated mice. Comparing gene expression of macrophages vs. monocytes from DSS mice revealed 44 genes (27 down- and 17 up-regulated) and 308 genes from naïve mice (196 down- and 112 up-regulated) were Log2-fold change ≥ 2 expressed (*P* < 0.01; Figure 3D). Once again there was a striking similarity independent of inflammation with 73/200 of the 100 largest absolute fold changing up- and down-regulated genes identical in steady state and DSS when comparing macrophage vs. monocyte gene expression. The genes most highly expressed in macrophages relative to monocytes in both health and inflammation were chemokines (Figures 3E,F). In particular macrophage mRNA expression of *Ccl7, Ccl8, Ccl12*, and *Cxcl1* was greatly elevated compared to monocytes, in both naïve and inflamed settings (Figure 3E). This suggests that in the colon, monocyte-derived macrophages rapidly develop the capability to produce chemokines that would facilitate recruitment of both their own monocyte precursors and neutrophils (39), with high level expression of these genes conserved in macrophages independent of inflammation. In addition, comparison of monocytes from DSS vs. naïve mice showed elevated expression of *Ccl7, Ccl8*, and *Cxcl1* upon inflammation (Figure 3E).

Surprisingly, *Ccl2* and *Ccl3*, which are crucial mediators of monocyte and macrophage recruitment, were not expressed at significantly different levels comparing monocytes to macrophages, either in the steady state or during inflammation (Figure 3E) (7, 9, 38, 40). In fact, levels of these transcripts were actually significantly reduced in both monocytes and macrophages when comparing inflamed to naïve settings (Figure 3E).

Having identified a distinctive chemokine signature in myeloid cells isolated from murine colitis, we next sought to assess whether a similar pattern of altered chemokine expression was evident during human colitis. Targeted colon biopsies from control, quiescent IBD and active IBD were analysed for mRNA expression of *CCL7, CCL8*, and *CXCL1* based on our murine array data. As in murine colitis, *CCL7, CCL8*, and *CXCL1* were all significantly increased in active IBD vs. quiescent IBD and controls (Figure 3G).

These data show the comparative effects of intestinal inflammation on the mRNA signatures of colon monocytes and macrophages, identifying altered expression in both macrophages and monocytes of genes that stabilise ribosome function during ER stress incurred by inflammation and chemokines involved in promoting monocyte recruitment incurred by monocyte-macrophage differentiation.

## Discussion

IBD is characterised by remitting relapsing inflammation of uncertain pathogenesis that in CD can occur anywhere in the GI tract and in UC occurs almost exclusively in the colon. However, existing data on the innate immune system in IBD has predominately focused on phenotyping immune cells from the small intestine, in isolation or a combination of small and large intestine, rather than the colon exclusively (2, 12, 14, 21, 22). Even within murine colitis models, the changes conferred by inflammation on the relative abundance of different myeloid cells and their relative frequency, proportion and cytokine responses are poorly described. Our study utilised both targeted biopsy material from a colon IBD cohort and a murine model of colitis, to show that inflammation dramatically altered the composition of the myeloid compartment in the colon.

Naïve colon monocytes quickly differentiate into resident macrophages after entering the intestine, a process that confers significant change in function towards tolerance of the microbiota (2). However, comparison of the gene expression changes that accompany the differentiation process in the steady state vs. colon inflammation, or indeed the expression differences of colon monocytes and macrophages before and after colitis, are unknown.

We observed that active human colon IBD was associated with significantly greater colon mRNA expression of *TNF, IL1B, IL6*, and ratios of CD14^Hi^:CD14^Lo^ and HLA-DR^Int^:HLA-DR^Hi^ cells vs. quiescent IBD and healthy controls. This is consistent with previous work that has reported an increase in CD14^Hi^ cells in inflamed intestinal mucosa that have features of less mature mononuclear cells, though our data is one of the first using an exclusive colon dataset of targeted biopsies comparing active and quiescent IBD to healthy controls (14). Indeed, identifying the role of intestinal CD14^Hi^ cells has been hampered somewhat by different methodologies (in particular differences in anatomical location sampling) and sub-classification in the literature (2, 11–14). As we were limited to assessment of healthy control sigmoid colon alone in our presented work, future studies will be required to address if IBD alters these populations to varying degrees in different regions of the colon.

CD14^Hi^HLA-DR^Hi^ cells from CD/UC surgical resection specimens may induce differentiation of naïve T cells into Th17 cells with a CD163^Lo^ subset able to produce greater IL-1β and IL-6 after TLR stimulation, compared to macro/microscopically normal areas from colon cancer resections (13). Similarly Kamada et al. reported an increase in IL-6, TNF, and IL-23 producing CD14^+^CD33^+^ cells in UC/CD surgical resection specimens compared to controls, with both Thiesen et al. (combined small and large intestine samples) and Magnusson et al. (discrete small and large intestine samples) reporting increased CD14^Hi^HLA-DR^Int^ cells in active vs. quiescent IBD and healthy controls (11, 12, 14). This highlights that increased numbers of CD14^Hi^ intestinal cells in the colon may be a reliable indicator of IBD severity.

Interrogation of a murine colitis model allowed us to accurately define the numerical and proportional changes in the colon myeloid compartment. Mirroring the increased colonic CD14^Hi^:CD14^Lo^ ratio observed in active human IBD, murine colitis was characterised by an increased Ly6C^Hi^CD64^+^ monocyte to Ly6C^−^CD64^+^F4/80^+^ macrophage ratio. Monocyte and neutrophil populations were the most sensitive (by fold change) to numerical expansion induced by DSS, with corresponding significant decreases in the proportion of macrophages and DCs within the myeloid compartment. Thus, one of the hallmarks of murine and human colitis is the accumulation of monocyte (mouse) or monocyte-like (human) cells, such that they undergo a large numerical increase after the onset of inflammation to represent a substantial proportion of the myeloid compartment compared to the resting state.

In addition to identifying this link between severity of colitis and increased tissue monocytes, we have shown for the first time the relative production of the key inflammatory cytokines IL-1β and TNF by intestinal myeloid cells in DSS colitis, revealing monocytes as the dominant IL-1β producing population. This was evident in both heightened per cell IL-1β production, and increased IL-1β^+^ monocyte numbers, compared to other myeloid cells. DSS did not increase per cell TNF production in any myeloid cell population. Therefore, in contrast to IL-1β, the increase in the number of TNF^+^ monocytes after DSS was due to increased recruitment and accumulation of monocytes, rather than increased per cell production of this cytokine. The importance of IL-1β producing intestinal monocytes for appropriate clearance of *Citrobacter* spp has recently been shown, where CCR2^+^ monocytes give rise to macrophages that induce IL-22 from ILC3 cells in an IL-1β dependent manner (38). Here we go further, having shown that during DSS colitis, macrophages, DCs and neutrophils in the colon can produce IL-1β in addition to monocytes.

In addition to assessment of tissue monocytes, we found that murine blood monocytes produced heightened IL-1β, but not TNF, during DSS colitis. This may indicate that intestinal inflammation confers systemic signals to blood monocytes increasing their IL-1β producing potential. In addition, neither IL1-β nor TNF were detectable in the absence of LPS stimulation, suggesting negligible levels of basal cytokine expression and that monocyte pro-inflammatory cytokines would only be released on tissue entry and TLR engagement. Whilst the specific systemic signals that might confer increased blood monocyte IL-1β potential are not yet known, it has recently been suggested that NK cell production of IFN-γ can influence monocyte progenitors in the bone marrow to adopt a regulatory phenotype during acute gastrointestinal infection, a process that precedes the onset of systemic inflammation (41). Indeed, granulocyte/monocyte adsorption (GMA) columns are currently being investigated as a therapy to reduce circulating CD14^+^CD16^+^ blood monocytes in patients with active IBD (16). Interestingly, those patients treated early with GMA display a more benign disease course, suggesting that acute rather than established inflammation is more amenable to monocyte intervention (42).

Whilst we have observed monocytes as a defining feature of both murine and human colon inflammation, it is important to note their macrophage-progeny are also thought to be important constituents of the repair process. For example, *Ccr2*^−/−^ mice sequester monocytes within the BM (43), resulting in depletion of tissue resident macrophages, reducing their susceptibility to acute DSS colitis but increasing susceptibility to chronic DSS mediated inflammation (7, 44). Conversely conditional depletion of CCR2^+^ monocytes using CCR2^DTR^ mice increases susceptibility to intestinal *Citrobacter* infection. This demonstrates the delicate monocyte-macrophage balance required to repel intestinal infection, limit potentially damaging monocyte recruitment and also resolve tissue damage. Therefore, strategies aiming to modulate pro-inflammatory monocytes during IBD may have a “window of opportunity” in acute disease that must be balanced against the importance of monocyte derived macrophages in resolving chronic intestinal inflammation and monocytes themselves in combatting opportunistic intestinal infection.

To identify the mechanisms that control monocyte and macrophage abundance and their role in colitis, we compared the mRNA profiles of purified colon monocytes and macrophages from DSS treated and control mice. DSS conferred similar changes to both monocyte and macrophage gene expression compared to the steady state. Pathway analysis revealed top dysregulated pathways in both populations were associated with ER stress and unfolded protein response (UPR) associated loci (*Eif3k, Rpl13a* down-regulated upon DSS and *Rpl31* and *Rpl37* up-regulated upon DSS). *Eif3k* for example encodes the k subunit of the eurkaryotic initiation factor 3 (eIF3) which is an important component of ER induced stress granules. Interestingly, the c subunit of eIF3 (*EIF3C*) has been identified as a risk susceptibility locus for IBD in early onset paediatric populations (45) and eIF3 protein is found at increased levels in the colonic mucosa of UC patients (46). This suggests decreased *Eif3k* mRNA expression in monocytes/macrophages in our data is part of a compensation mechanism for increased ER stress in these populations during colitis (46). The other major down-regulated gene, *Rpl13a*, encodes a ribosomal protein that forms part of an IFN-γ activated inhibitor of translation (GAIT) complex that binds untranslated regions of several cytokine mRNAs during inflammation. Myeloid specific knockout of *Rpl13a* renders the host susceptible to LPS-induced endotoxaemia and DSS colitis (47, 48). *Rpl13* deficient macrophages also display an unregulated expression of CCL8, suggesting that the *Rpl13a* down-regulation we observed in intestinal macrophages is part of the host response to the barrier breakdown caused by DSS that would permit increased recruitment of cytokine producing inflammatory monocytes (47). The function of other ribosomal proteins in mediating macrophage/monocyte responses during colitis is not well-characterised. However, *Rpl31* and *Rpl37*, expression of which were greatly increased by macrophages/monocytes during DSS inflammation, have been implicated in cell cycle in bacteria and cancer, and preventing p53 mediated apoptosis (49–51). Therefore, increased expression of *Rpl31/37* and UPR transcripts by monocytes/macrophages after DSS administration may permit these cells to operate in hostile environments by facilitating protein translation and maintaining key cellular processes for survival.

Comparison of mRNA expression changes between colon macrophage and monocytes revealed a highly conserved set of genes that was independent of DSS-elicited inflammation. A range of monocyte and neutrophil attracting chemokines (*Ccl7, Ccl8, Cxcl1*) were more highly expressed in macrophages compared to monocytes from both steady state and DSS colitis. In addition, expression of these chemokine transcripts were elevated in monocytes from DSS treated vs. naïve mice. This shows that expression of these chemokine transcripts was globally elevated upon colitis, a feature that was also evident when comparing human colon samples from active vs. quiescent IBD and controls. There are numerous chemokines (CCL2, CCL7, CCL8, and CCL12) that have been reported to recruit CCR2^+^ classical monocytes (7, 9). Of these, CCL2 is known to be an important ligand for CCR2, though its role in colitis is unclear, as DSS treated *Ccl2*^−/−^ mice have been shown to develop either exacerbated or ameliorated colitis (52, 53). In contrast to other monocyte chemokines, we found that *Ccl2* expression was equivalent between monocytes and macrophages. However, interestingly, *Ccl2* expression was actually reduced in both monocytes and macrophages when comparing populations before and after DSS. This suggests that macrophages and monocytes are not the key source of CCL2 release during colitis. The importance of CCL7, CCL8, CCL12, and CXCL1 to mediate colitis inflammation, and the consequence of enhanced monocyte expression of these chemokines on pathology, is not known. Although it has been reported that CD169^+^ macrophages express *Ccl2, Ccl7*, and *Ccl8* during colitis (the contribution of monocytes was not considered) (9, 54), and analysis of colon IBD sections has suggested CCL8 as the greatest up-regulated chemokine released from inflammatory cells (55). Therefore, as our study directly compare transcriptomes of intestinal monocytes and macrophages in both naïve and inflamed settings, we propose that colon macrophages in colitis are important sources of CCL7/8 and that, in addition, monocytes in inflamed settings possess an increased ability to express *Ccl7/8* that enables further monocyte entry into inflamed tissue sites.

Together, our murine and human data suggest that monocyte recruitment, and systemic priming of monocyte IL-1β release, are key components involved in escalation of colon inflammation in colitis. As such, targeted interference of monocyte recruiting chemokines like *CCL7, CCL8*, or *CXCL1* may help preserve the steady state monocyte:macrophage balance that is disrupted in pathological inflammatory states, such as IBD. Elegant tracking studies have shown adult murine colon macrophages develop from Ly6C^Hi^CCR2^+^ blood monocytes, to Ly6C^+^CD11b^+^ colon monocytes, to mature F4/80^+^CX3CR1^Hi^ macrophages, a process that is also characterised by a dramatic change in function and phenotype as described above (2, 8, 10). Thus, modulation of either monocyte or macrophage representation will likely change the pro-inflammatory capacity of the GI tract.

## Conclusion

In summary, we have shown accumulation of pro-inflammatory monocytes in both murine colitis and human IBD, and suggest that targeting these monocytes, or their recruitment by macrophages, may represent a new and exciting future therapy for intestinal inflammation.

## Author contributions

G-RJ and PC: study concept and design, acquisition of data, analysis and data interpretation, drafting of the manuscript, critical revision of the manuscript for important intellectual content; AK, TF, SB, and CB: acquisition of data, drafting of the manuscript, critical revision of the manuscript for important intellectual content; AI: analysis and data interpretation, drafting of the manuscript, critical revision of the manuscript for important intellectual content; AM and MT: conception and design of the study, data interpretation, critical revision of the manuscript for important intellectual content. All authors approved the final manuscript.

### Conflict of interest statement

The Manchester Collaborative Centre for inflammation research is a joint venture between the University of Manchester and GSK. The authors declare that the research was conducted in the absence of any commercial or financial relationships that could be construed as a potential conflict of interest.

## Supplementary material

Supplementary Figure 1 **(A)** Representative gating strategy by flow cytometry to identify Live/singlet/Lineage^−^/CD45^+^HLA-DR^+^ cells from colon lamina propria. **(B)** The cellular component of blood was isolated from day 6 DSS treated or drinking water controls and stimulated with 1 μg/ml LPS and GolgiStop 1 μl/ml for 3 h. CD11b^+^CD68^+^Ly6C^Hi^ blood monocytes were identified by flow cytometry and assessed for the expression of TNF by intracellular staining, *n* = 12–15 mice per group analysed by linear regression of three independent experiments.

Supplementary Table 1 qPCR colon biopsy sample patient demographics. Twelve healthy control, 12 quiescent IBD, and 19 active IBD colon biopsy specimens from 8 healthy control, 11 quiescent IBD, and 17 active IBD patients attending for colonoscopy were obtained for RNA extraction and RT-qPCR analysis. Patient records were reviewed for demographic, IBD location and disease behaviour by Montreal classification. IM, Immunomodulatory drugs; MTX, methotrexate; ADA, adalimumab; IFX, infliximab; 6′MP, 6-mercaptopurine; 5′ASA, 5′aminosalicylic acid. Montreal classification for CD: A1, age at onset < 16 years; A2, between 17 and 40 years; A3, >40 years. L1, ileal disease location; L2, colonic disease location; L3, ileocolonic disease location. B1, inflammatory phenotype; B2, stricturing phenotype; B3, penetrating phenotype. Montreal classification for UC: E1, proctitis; E2, rectosigmoid disease; E3, disease proximal to the splenic flexure. CD, Crohn's disease; UC, ulcerative colitis. # Patient ID is the identifier allocated to each individual providing tissue, such that donors that gave more than 1 sample can be identified.

Supplementary Table 2 Flow cytometry antibody details.

Supplementary Table 3 qPCR Primer details.
