# Supplementary figures and images for: Dynamics of Colon Monocyte and Macrophage Activation During Colitis
Source: Front Immunol. 2018 Nov 27;9:2764. doi: 10.3389/fimmu.2018.02764 (PMC6277765; doi:10.3389/fimmu.2018.02764)

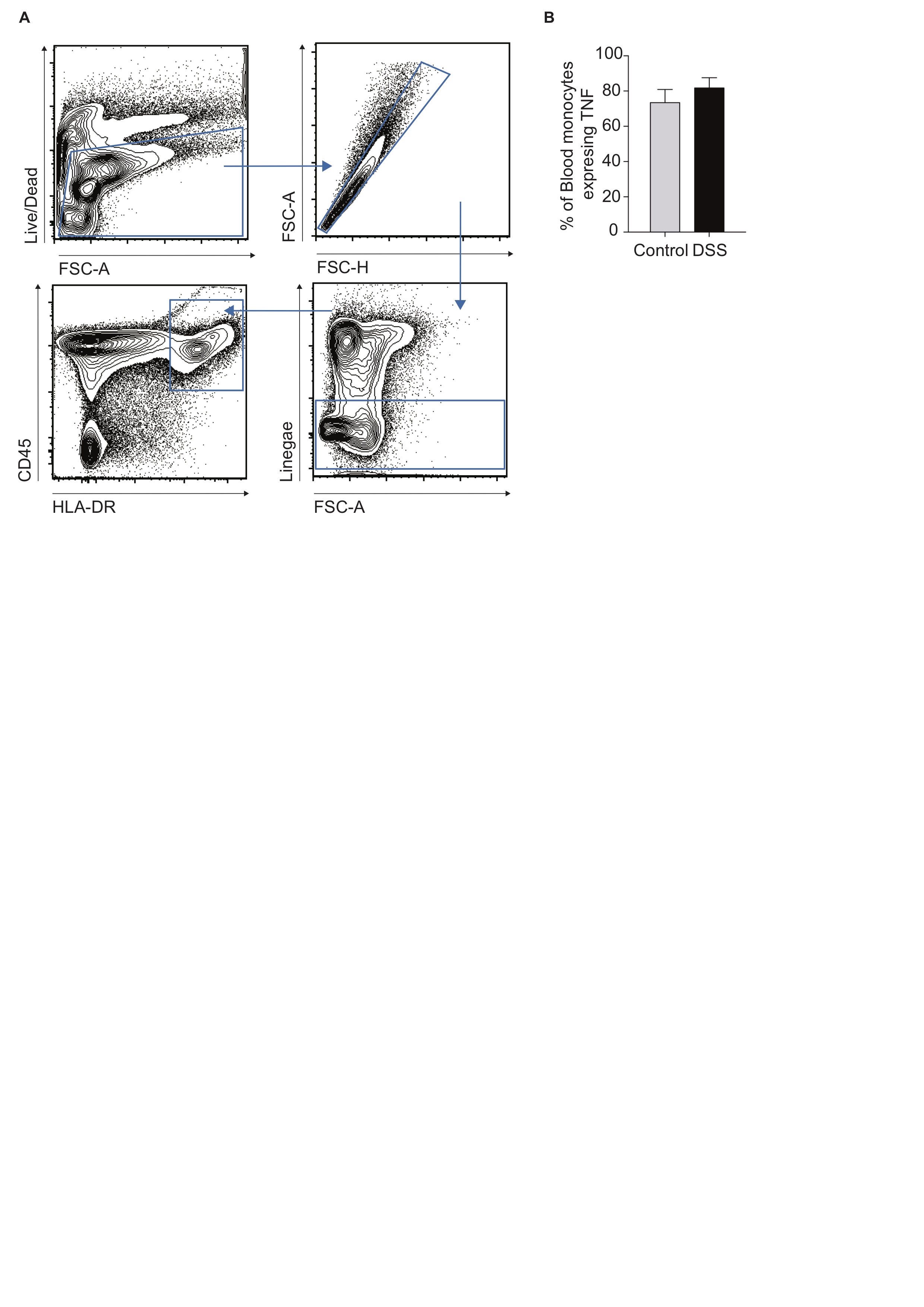

Supplement: Supplementary Figure 1 — (A) Representative gating strategy by flow cytometry to identify Live/singlet/Lineage−/CD45+HLA-DR+ cells from colon lamina propria. (B) The cellular component of blood was isolated from day 6 DSS treated or drinking water controls and stimulated with 1 μg/ml LPS and GolgiStop 1 μl/ml for 3 h. CD11b+CD68+Ly6CHi blood monocytes were identified by flow cytometry and assessed for the expression of TNF by intracellular staining, n = 12–15 mice per group analysed by linear regression of three independent experiments. [file Image_1.JPEG]
